# 3,5-Dicaffeoyl-Epi-Quinic Acid Isolated from Edible Halophyte* Atriplex gmelinii* Inhibits Adipogenesis via AMPK/MAPK Pathway in 3T3-L1 Adipocytes

**DOI:** 10.1155/2018/8572571

**Published:** 2018-11-21

**Authors:** Jung Hwan Oh, Jung Im Lee, Fatih Karadeniz, Youngwan Seo, Chang-Suk Kong

**Affiliations:** ^1^Department of Food and Nutrition, College of Medical and Life Sciences, Silla University, Busan 46958, Republic of Korea; ^2^Division of Marine Bioscience, College of Ocean Science and Technology, Korea Maritime and Ocean University, Busan 49112, Republic of Korea; ^3^Marine Biotechnology Center for Pharmaceuticals and Foods, College of Medical and Life Sciences, Silla University, Busan 46958, Republic of Korea; ^4^Department of Convergence Study on the Ocean Science and Technology, Ocean Science and Technology School, Korea Maritime and Ocean University, Busan 49112, Republic of Korea

## Abstract

*Atriplex gmelinii* is an edible halophyte that has been suggested to possess various health benefits. In the present study, 3,5-dicaffeoyl-epi-quinic acid (DEQA) isolated from* A. gmelinii* was tested for its ability to prevent adipogenesis in 3T3-L1 cells. Also, the molecular mechanisms by which DEQA affects differentiation of 3T3-L1 cells were investigated. The introduction of DEQA to differentiating 3T3-L1 preadipocytes resulted in suppressed adipogenesis and lowered expression of adipogenesis-related factors, PPAR*γ*, C/EBP*α*, and SREBP-1c. Treatment of 3T3-L1 adipocytes with DEQA notably decreased the levels of phosphorylated p38, ERK, and JNK. In addition, presence of DEQA upregulated the levels of both inactive and phosphorylated adenosine monophosphate-activated protein kinase (AMPK) and its substrate, acetyl-CoA carboxylase (ACC). Taken together, current results indicated that DEQA exhibited a significant antiadipogenesis activity by activation of AMPK and downregulation of MAPK signal pathways in 3T3-L1 preadipocytes.

## 1. Introduction

Obesity is a worldwide health problem and many diseases such as cardiovascular disease, liver and kidney diseases, diabetes, and cancer are considered to be linked with the prior cases of excess body fat accumulation [1–3]. Obesity development occurs through high rates of proliferation and differentiation of white adipocytes, which result in expanding white adipose tissue. Negating the effects of obesity while elucidating the underlying mechanism and preventing the formation of adipose tissue have been of great interest in pharmaceutical field.

Adipose tissue that stores the excessive fat in the body plays pivotal roles in controlling body homeostasis [4]. It also secretes hormones and interacts with regulation of several important organs. Current studies revealed the role of adipocyte dysfunction in the progression of obesity, cardiovascular diseases, and various metabolic diseases including type 2-diabetes [5–7]. Formation of new adipocyte cells, adipogenesis, takes place in two stages which can be called the determination and differentiation phases. The change of mesenchymal stem cells into adipocyte lineage is called the determination stage while the decisive differentiation of these preadipocytes into mature adipocytes is regarded as the differentiation stage [8].

During decisive adipocyte differentiation, the peroxisome proliferator-activated receptor *γ* (PPAR*γ*), sterol regulatory element-binding protein 1c (SREBP-1c), and CCAAT-enhancer-binding protein *α* (C/EBP*α*) transcription factors are expressed progressively and increasingly, triggering further pathways for adipogenesis [9]. Notably, PPAR*γ* is a nuclear receptor which has been studied in detail over the years and has been accepted as the key regulator in adipogenesis. PPAR*γ* activation is compulsory and often enough for adipogenesis and expression of adipocyte characteristics such as fat storage and related hormone secretion [10]. PPAR*γ* and C/EBP*α* pathways activate and/or are regulated by different metabolic pathways such as mitogen-activated protein kinase (MAPK) and 5′ adenosine monophosphate-activated protein kinase (AMPK) that are required to maintain cellular functions as adipocytes [11–13].

*Atriplex gmelinii* is a halophyte growing natively in Korea, Japan, and North America [14]. Leaves and flowers of* A. gmelinii* are referred in traditional medicine sources and utilized in local diet. Caffeoylquinic acid (CQA) is a phenylpropanoid found in variety of sources including but not limited to coffee beans, sweet potato, propolis and other dietary sources [15]. CQA derivatives have been credited for sets of bioactivities such as antioxidant, antibacterial, anticancer, antihistamic, and other biological effects [16–18]. In this study, a CQA derivative, 3,5-dicaffeoyl-epi-quinic acid (DEQA) from* A. gmelinii* was isolated and its antiobesity activity was tested in 3T3-L1 preadipocyte cells.

## 2. Materials and Methods

### 2.1. Chemicals and Materials

Dulbecco's modified Eagle's medium (DMEM) and fetal bovine serum (FBS) were acquired from Gibco BRL (Grand Island, NY). Immunoblotting antibodies were procured from Cell Signaling Technology (Danvers, MA, USA). Primers for reverse transcription polymerase chain reaction was purchased from Bio-RAD (Hercules, CA, USA). Remaining materials and chemicals were ordered from Sigma–Aldrich (St. Louis, MO, USA) unless noted.

### 2.2. Plant Material

The sample (3 kg) of* A. gmelinii* was air-dried and cut into small pieces prior to maceration. Ground samples were extracted twice with methylene chloride (CH_2_Cl_2_) for 24 h at room temperature. The extraction solution was concentrated in vacuo, and crude extract was obtained. Remaining ground samples were then subjected to another extraction with methanol (MeOH), using the same procedure as above. The combined crude extracts from CH_2_Cl_2_ and MeOH extraction were partitioned between methylene chloride and water (H_2_O). The methylene chloride layer was concentrated to dryness under reduced pressure and the obtained residue was repartitioned between* n*-hexane and 85% aqueous MeOH. The aqueous layer was also concentrated to dryness and further partitioned between* n*-BuOH and H_2_O. The *n*-BuOH fraction was further subjected to isolation procedures and yielded the bioactive compound DEQA. The compound was identified as DEQA by extensive 2-D NMR experiments and comparison with data reported in the literature. DEQA was dissolved in methanol and diluted with DMEM.

### 2.3. Cell Culture and Adipocyte Differentiation

Mouse 3T3-L1 preadipocytes were cultured in DMEM supplemented with 10% fetal bovine serum (FBS) at 37°C with 5% CO_2_ in a humidified atmosphere. At the second day of postconfluence, differentiation of the preadipocytes was induced with a differentiation mixture containing methylisobutylxanthine (0.5 mM), dexamethasone (0.25 *μ*M), and insulin (5 *μ*g/ml) in culture medium. The differentiation medium was replaced with feeding medium containing DMEM with10% FBS and insulin only (5 *μ*g/ml) after 48 h incubation. This medium was replaced with fresh one every 2 days until the maturation of adipocytes was confirmed by lipid droplet observation under a microscope. DEQA was administered to the cell culture medium starting with differentiation medium and included in all medium changes. Cytotoxicity level of DEQA to 3T3-L1 cells was evaluated by MTT assay as previously described [19].

### 2.4. Oil-Red O Staining of the Lipid Droplets

Intracellular lipid accumulation of 3T3-L1 adipocytes were observed by Oil-Red O staining of the triglycerides. Cell culture medium was removed, and cells were washed with phosphate buffer saline (PBS). Following the removal of PBS from wells, cells were fixed on wells with 3.7% fresh formaldehyde in PBS and left at room temperature for 1 h. Oil-Red O (0.5% w/v) solution (60% isopropanol and 40% water) was filtered and applied to the cells. After staining incubation of 1 h, the Oil-Red O solution was aspired from the plates. Images of cells with lipid droplets were collected through a Nikon Instruments microscope (Tokyo, Japan). Oil-Red O stain from intracellular lipids was collected with 100% isopropanol and quantified by optical absorbance measurement at 500 nm using a microplate reader (Tecan Austria GmbH, Austria).

### 2.5. Reverse Transcription Polymerase Chain Reaction Analysis

Total RNA was isolated from 3T3-L1 adipocytes treated with/without DEQA using Trizol reagent (Invitrogen Co., CA, USA). Synthesis of cDNA was started with addition of total RNA (2 *μ*g) to RNase-free water containing oligo (dT) and followed by denaturation at 70°C for 5 min and cooling immediately. Next, the mixture was reverse transcribed in a master mix (1X RT buffer, 1mM dNTPs, 500 ng oligo (dT), 140 U M-MLV reserve transcriptase, and 40 U RNase inhibitor) using an automatic T100 Thermal Cycler (Bio-Rad, UK) with a cycle of 42°C for 60 min and 72°C for 5 min. Following sense and antisense primers were used for the amplification of target cDNA: forward 5′-TTT-TCA-AGG-GTG-CCA-GTT-TC-3′ and reverse 5′-AAT-CCT-TGG-CCC-TCT-GAG-AT-3′ for PPAR*γ*; forward 5′-TGT-TGG-CAT-CCT-GCT-ATC-TG-3′ and reverse 5′-AGG-GAA-AGC-TTT-GGG-GTC-TA-3′ for SREBP-1c; forward 5′-TTA-CAA-CAG-GCC-AGG-TTT-CC-3′ and reverse 5′-GGC-TGG-CGA-CAT-ACA-GTA-CA-3′ for C/EBP*α*; forward 5′-CCA-CAG-CTG-AGA-GGG-AAA-TC-3′ and reverse 5′-AAG-GAA-GGC-TGG-AAA-AGA-GC-3′ for *β*-actin. The cDNA amplification was carried out using T100 Thermal Cycler (Bio-Rad, UK) with cycle settings at 95°C for 45 sec, 60°C for 1 min and 72°C for 45 sec for 30 cycles. Final PCR products were separated by gel electrophoresis on 1.5% agarose gel for 30 min at 100 V. Gels were then stained with 1 mg/ml ethidium bromide and visualized by UV light using Davinch-Chemi imager™ (CAS-400SM, Seoul, Korea).

### 2.6. Protein Immunoblotting

Protein immunoblotting was performed with standard Western blotting procedures. Concisely, cells were lysed in RIPA lysis buffer (Sigma–Aldrich Corp., St. Louis, MO, USA) at 4°C for 30 min. The acquired cell lysate (25 *μ*g) was used for Western blot analysis. Proteins in cell lysate were separated by sodium dodecyl sulphate-polyacrylamide gel electrophoresis (SDS-PAGE) on 4% stacking and 12% separating gels. Proteins subjected to separation were then electrotransferred to a polyvinylidene fluoride membrane (Amersham Pharmacia Biotech., England, UK), which was blocked with 5% skim milk powder in TBST buffer after transfer. The membrane was hybridized with primary antibodies diluted (1:1000) in primary antibody dilution buffer containing 1X TBST with 5% bovine serum albumin at 4°C overnight and incubated with horseradish-peroxidase-conjugated secondary antibody at room temperature for 2 h. Immunoreactive proteins bands were visualized by a luminol-based chemiluminescence assay kit (Amersham Pharmacia Biosciences, England, UK) according to the manufacturer's instructions. Images of protein bands were captured using a Davinch-Chemi imager™ (CAS-400SM, Seoul, Korea).

### 2.7. Statistical Analysis

The data was presented as mean of three independent experiments ± SD. Statistically significant differences between the means of the individual groups were determined by one-way analysis of variance (ANOVA) followed by Duncan's multiple range tests using SAS v9.1 software (SAS Institute, Cary, NC, USA). The significance of differences was defined at the* p*<0.05 level.

## 3. Results

**Figure 1:**
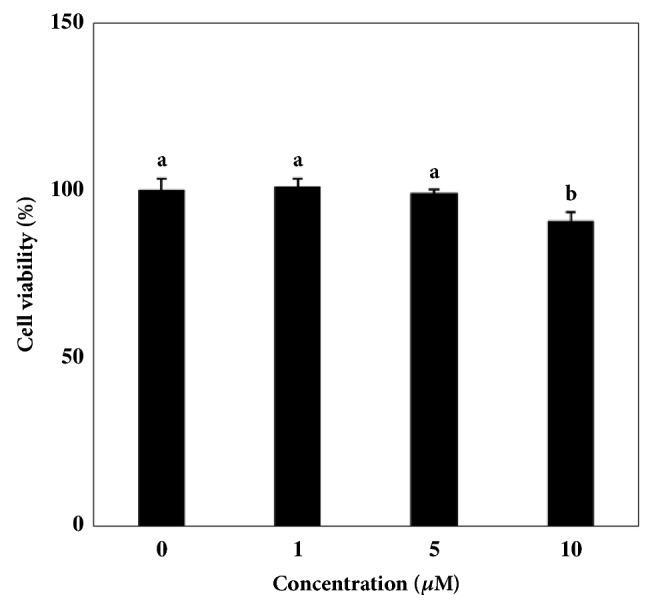
Effect of DEQA on the viability of 3T3-L1 cells. Cytotoxicity of DEQA in 3T3-L1 cells was observed by MTT assay. Cell viability following DEQA treatment was evaluated by their ability to form MTT-Formazan crystals and measured by the absorbance values at 540 nm. Viability of the cells was quantified as a percentage of untreated control. Values are means±SD (*n*=3).  ^a-b^Means with the different letters are significantly different (*p*<0.05) by Duncan's multiple range test.

Prior to assays regarding the ability of DEQA hindering differentiation and adipocyte profile of 3T3-L1 preadipocytes, its biocompatibility was tested by MTT assay. At treated concentrations (1, 5 and 10 *μ*M) DEQA was observed to be nontoxic to preadipocytes compared to untreated cell viability after 48 h incubation (Figure 1). Future assays were carried out using these concentrations.

### 3.1. DEQA Inhibits Lipid Accumulation in Differentiated 3T3-L1 Adipocytes

**Figure 2:**
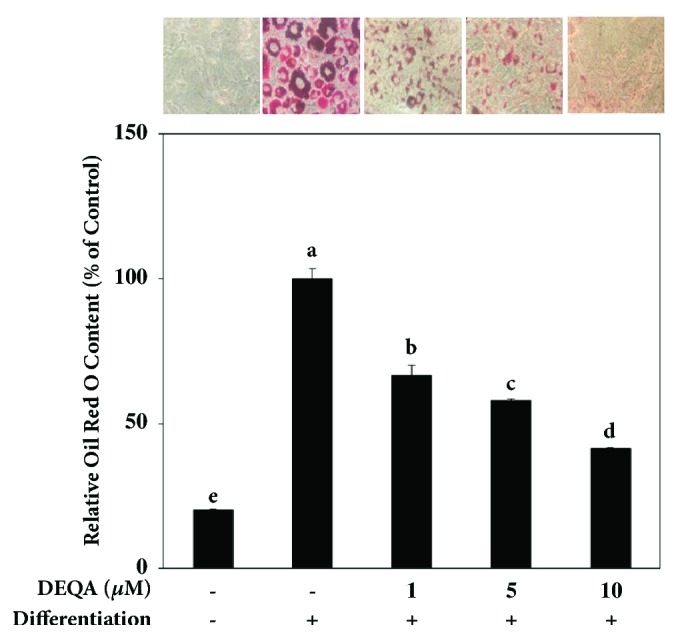
Effect of DEQA on the intracellular lipid accumulation of the differentiated 3T3-L1 cells. Cells were induced to adipogenesis with a differentiation mixture with different concentrations of DEQA. Following the successful differentiation of the cells, intracellular lipid droplets were stained by Oil-Red O staining and images were taken. Lipid accumulation was measured by the absorbance values of the eluted stain from cells at 500 nm. Accumulated lipid droplets were quantified as a percentage of the fully differentiated untreated control cells. Values are means±SD (*n*=3).  ^a-e^Means with the different letters are significantly different (*p*<0.05) by Duncan's multiple range test.

To determine the ability of DEQA to prevent adipogenic differentiation, DEQA was introduced to the differentiating 3T3-L1 preadipocytes. Successful differentiation of cells into mature adipocytes was confirmed by staining the lipid droplets as an indicator for fat storage ability. Cell images showed that the differentiated 3T3-L1 adipocytes accumulated high amounts of intracellular lipid droplets compared to nondifferentiated preadipocytes (Figure 2). Treatment with DEQA also lowered the amount of these lipid droplets as it was seen in the Figure 2a, in a dose-dependent manner. Staining of lipid droplets was quantified by elution of the Oil-Red O stain that was bind to the lipid droplets. Expectedly, DEQA was able to hinder the fat storing ability of differentiated adipocytes compared to untreated control cells (Figure 2). At the concentration of 10 *μ*M, amount of triglyceride droplets in cells was 41.50% of the control adipocytes. This decreasing effect was 66.73% and 58.05% of the control cells at the concentrations of 1 and 5 *μ*M, respectively.

### 3.2. DEQA Inhibits the Expression of Adipogenesis-Specific Key Transcription Factors

**Figure 3:**
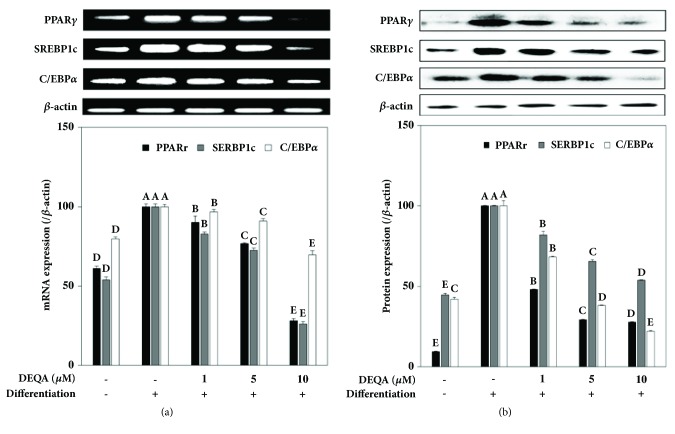
Effect of DEQA on the expression of key adipogenesis transcription factors; PPAR*γ*, SREBP-1c, and C/EBP*α*. (a) Effect of DEQA on the mRNA levels of the transcription factors analyzed by RT-PCR. Regulation of the mRNA levels was quantified by the density of the bands and normalized against the housekeeping gene *β*-actin. Effect of DEQA on mRNA levels was given as the percentage of the fully differentiated untreated control cells. (b) Effect of DEQA on the protein levels of the transcription factors analyzed by Western blotting. The protein levels were quantified by the density of the bands and normalized against the housekeeping protein *β*-actin. Effect of DEQA on protein levels was given as the percentage of the fully differentiated untreated control cells. Values are means±SD (*n*=3).  ^A-E^Means with the different letters are significantly different (*p*<0.05) by Duncan's multiple range test.

The effect of DEQA on adipogenesis-specific key transcription factors was analyzed by reverse transcription polymerase chain reaction and immunoblotting for gene and protein expression, respectively. DEQA was introduced to 3T3-L1 cells during the differentiation period and its effect on the maturation into adipocytes was confirmed. The mRNA levels of PPAR*γ*, SREBP-1c, and C/EBP*α* were all increased in the mature adipocytes compared to nondifferentiated preadipocytes (Figure 3(a)). Treatment with DEQA decreased the mRNA levels of transcription factors in a significant level starting from the lowest dose (1 *μ*M) introduced. In nondifferentiated preadipocytes, level of PPAR*γ* mRNA was 61% of mature adipocytes. DEQA lowered PPAR*γ* mRNA by 9.8%, 23.3%, and 71.8% compared to control adipocytes at the concentrations of 1, 5, and 10 *μ*M, respectively. Similar inhibition patterns were observed for both SREBP-1c and C/EBP*α*. At 10 *μ*M, DEQA treated cells exhibited 26.1% of SREBP-1c and 69.8% of C/EBP*α* of what control cells expressed. To see the effect of DEQA on protein expression of these key transcription factors, protein levels were observed by Western blotting. Inhibition pattern was of similar to mRNA expression inhibition. Maturation into adipocytes resulted in elevated amounts of PPAR*γ*, SREBP-1c, and C/EBP*α* proteins. Introduction of DEQA into differentiation process prevented elevation of these transcription factors and exhibited lower protein levels. Amounts of PPAR*γ*, SREBP-1c, and C/EBP*α* were 72.2%, 46.0%, and 77.8% lower than those of control adipocytes following the treatment of 10 *μ*M DEQA (Figure 3(b)). As in mRNA levels, DEQA showed its effect in a dose-dependent manner. Inhibition of key transcription factors necessary for adipocyte differentiation and maintaining adipocyte profile indicated DEQA prevents preadipocytes which undergo adipogenesis differentiation when introduced during differentiation process. Presence of DEQA prevented preadipocytes from responding to the differentiation stimuli and transcription factors which, therefore, were expressed in notably lower amounts compared to untreated fully differentiated control adipocytes.

### 3.3. DEQA Regulates the MAPK and AMPK Pathways of Adipogenesis

**Figure 4:**
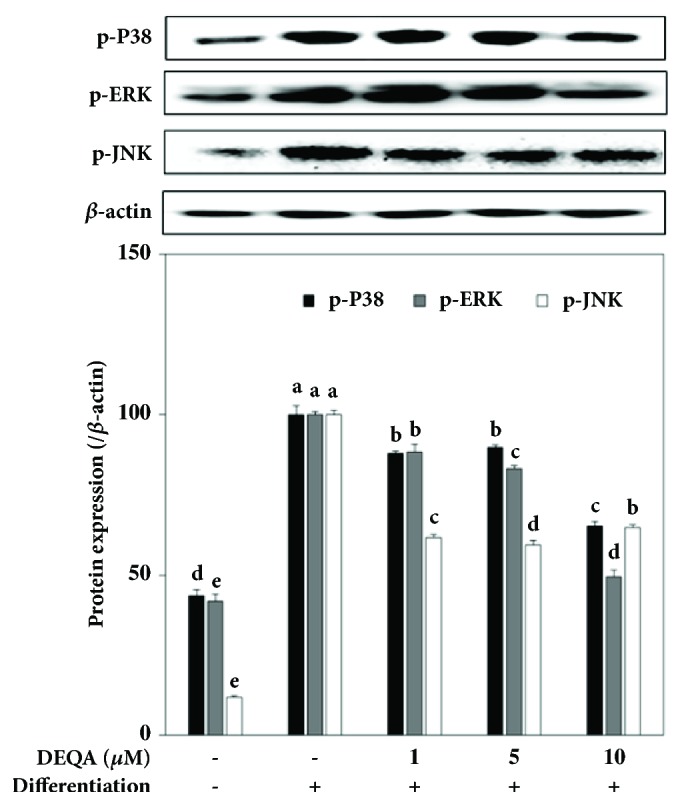
Effect of DEQA on the protein levels of the phosphorylated (p-) MAPK pathway proteins p38, ERK, and JNK analyzed by Western blotting. The protein levels were quantified by the density of the bands and normalized against the housekeeping protein *β*-actin. Effect of DEQA on protein levels was given as the percentage of the fully differentiated untreated control cells. Values are means±SD (*n*=3).  ^a-e^Means with the different letters are significantly different (*p*<0.05) by Duncan's multiple range test.

In order to have an insight on the action mechanism of DEQA-linked adipogenesis inhibition, crucial MAPK and AMPK pathway protein levels were analyzed. Phosphorylation of the MAPK pathway proteins p38, ERK, and JNK was observed by immunoblotting of the correspondent proteins. Differentiation of the 3T3-L1 cells accompanied by elevated activation of the MAPK as the phosphorylated p38 (p-p38), ERK (p-ERK), and JNK (p-JNK) protein levels was 56.4%, 58.2%, and 89.2% higher in mature 3T3-L1 adipocytes than that of nondifferentiated preadipocytes (Figure 4). Treatment with 1, 5, and 10 *μ*M DEQA lowered the phosphorylated protein levels of these MAPK pathway proteins. At the concentration of 10 *μ*M DEQA, p-p38 levels were 65.0% of the untreated control adipocytes while p-ERK amount was 49.5% and p-JNK amount was 64.9% of control.

**Figure 5:**
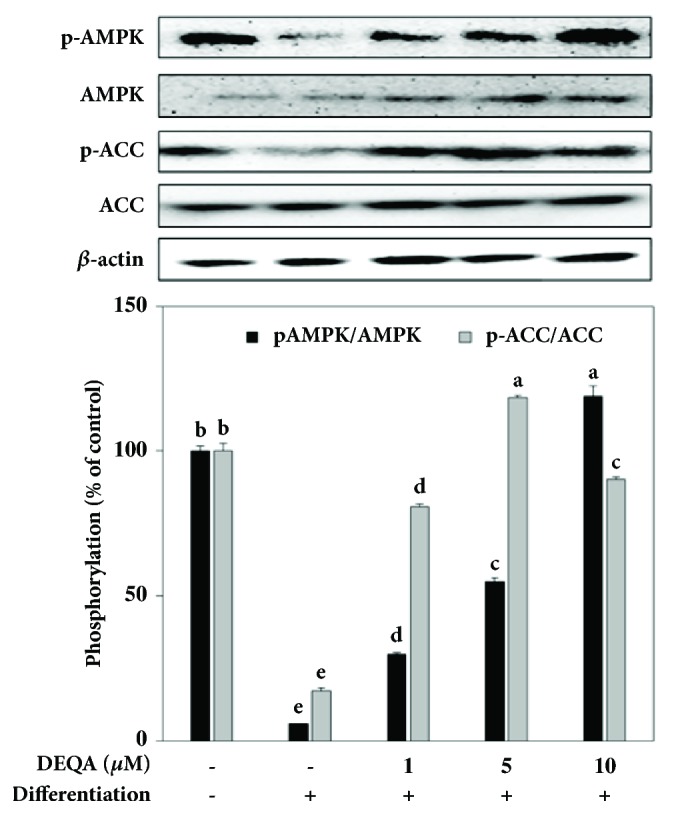
Effect of DEQA on the protein levels of the inactive and phosphorylated (p-) AMPK and its substrate ACC analyzed by Western blotting. The protein levels were quantified by the density of the bands and normalized against the housekeeping protein *β*-actin. Regulatory effect of DEQA on the phosphorylation of AMPK and ACC was given as the percentage of the nondifferentiated untreated control cells evaluated by the fold change of phosphorylated protein to inactive protein. Values are means±SD (*n*=3).  ^a-e^Means with the different letters are significantly different (*p*<0.05) by Duncan's multiple range test.

In addition, the inactivated and phosphorylated levels of AMPK and related protein ACC were analyzed with or without DEQA treatment to evaluate the samples' efficiency in terms of energy expenditure in adipocytes as an indicator of an effect against lipid accumulation of the differentiating cells at the terminal differentiation process. Differentiation of 3T3-L1 adipocytes resulted in suppressed levels of p-AMPK while the inactive AMPK levels stayed statistically same (Figure 5). Same pattern of suppression was observed for the phosphorylation of ACC. However, treatment with DEQA regulated the suppressed phosphorylation of AMPK and ACC seen as a dose-dependent increase in the protein levels of p-AMPK and p-ACC compared to untreated control group. In addition, nonphosphorylated AMPK and ACC levels were also observed to be elevated by DEQA treatment. At the concentration of 10 *μ*M DEQA, the phosphorylation of AMPK and ACC was elevated 20.50- and 5.25-fold, respectively, in comparison with control adipocyte group.

## 4. Discussion

Obesity has long become a worldwide health threatening disorder rather than a simple aesthetic problem, linked with several metabolic disorders, cardiovascular diseases, and cancer [1]. At the cellular level, elevated proliferation and formation of adipocytes, building blocks of adipose tissue, are the main character of obesity progression [3]. Preventing and treating obesity has been going on with several approaches on different mechanisms of actions such as balancing energy homeostasis, decreasing adipocyte proliferation, stimulating adipocyte apoptosis, elevated lipolysis, and suppressed adipocyte differentiation [20].

Edible plants that have been referred in traditional medicinal sources and are part of local diet are sought after for natural product studies due to their various benefits such as few side effects and high biocompatibility. Their bioactive constituents were reported to be able to treat, prevent, or cure numerous disorders and so research on the utilization of these compounds in pharmaceutical and nutraceutical fields is now attracting great interest. Halophytes and their active compounds have many pharmacological health benefits, including liver protection, antioxidative, anticancer, antimicrobial, antiatherogenic, antiallergic, and anti-inflammatory [21, 22].* A. gmelinii* is an edible halophyte native to Korea, Japan, and North America. It has been a part of diet on some regions and referred in traditional medicine sources. Although some studies reported its antioxidative effect [19], which is common in halophytes due to their high polyphenol content, literature lacks any further studies regarding its potential bioactivities. CQA is a common component of several edible plants consumed all over the world that also possess antioxidant properties [15]. Derivatives of CQA were credited to act against several health conditions such as cancer, diabetes, inflammation, oxidative stress, and obesity [15, 23, 24]. A derivative of CQA, 3,5-dicaffeoyl-epi-quinic acid (DEQA), was isolated from* A. gmelinii* as a part of ongoing study to reveal the bioactive effects of* A. gmelinii* and its bioactive constituents. DEQA is a potent phenolic that is found in a wide variety of plants that has been referred in several traditional medicine books. Studies showed that DEQA and its close derivatives have many beneficial effects on human health, including cardiovascular protection, anticancer activity, antiallergy activity, antiviral activity, and anti-inflammatory effects. However, to the best of our knowledge, there is no report that presents effect or action mechanism of DEQA on adipogenesis.

In this study, we carried out bioactivity screening and mechanism elucidation experiments targeted at identifying whether DEQA can prevent adipocyte differentiation in 3T3-L1 preadipocytes and if so what is the mechanism of the antiobesity activity of DEQA. The treatment of 3T3-L1 preadipocytes with DEQA regulated their differentiation in a dose-dependent manner (Figure 2). The inhibition of adipocyte differentiation is activated and controlled by a complex but well-studied network of transcription factors and adipogenesis-related enzymes. PPARs are ligand-activated transcription factors and known to take crucial parts in glucose metabolism [10]. Especially PPAR*γ* strongly regulate the differentiation of preadipocytes into adipocytes and maintaining the adipocyte profile. In addition to PPARs, C/EBPs are important proteins in the pathway that progress and perpetuate the final differentiation of adipocytes [9]. At the start of adipogenesis, the activation of PPAR*γ* follows the activation of C/EBP*α*, which sequentially progress differentiation of adipocytes and regulate insulin sensitivity in mature adipocytes. During these adipocyte differentiation steps, another transcription factor, SREBP-1c, is associated with fatty acid metabolism which in turn regulates the storage of fat in differentiated adipocytes [25]. Consequent PPAR*γ*, C/EBP*α*, and SREBP-1c activation is known to strictly control the differentiation of preadipocytes and marks the successful maturation into adipocytes and storing lipids.

Current results revealed that DEQA treatment considerably downregulated PPAR*γ*, C/EBP*α*, and SREBP-1c expression at both mRNA and protein level, which is vital for differentiation. Complementary to current results, CQA derivatives that inhibit adipogenesis show their efficiency by suppressing the PPAR*γ*-linked activation of aforementioned proteins. Several other inhibitors of adipogenic differentiation such as KLF2 and calcineurin [26] were also aimed to intervene with the activation of C/EBP*α* and PPAR*γ*, the pivotal members of transcriptional regulation of adipogenesis. Therefore, these findings demonstrate that treatment with DEQA suppresses the elevated mRNA and protein expressions of C/EBP*α*, PPAR*γ*, and SREBP-1c, by that preventing the differentiation of preadipocytes into adipocytes (Figure 3).

The key MAPK pathway proteins, namely, p38, ERK, and JNK, are argued to be significantly involved in adipogenesis [11]. Reduced differentiation of adipocytes was achieved through the downregulation of p38 and ERK by inhibitors, suggesting that these proteins play essential roles in adipogenesis. Although JNK is also speculated to be a part of this regulation, there is minimal evidence for the role that JNK plays in adipogenesis. A previous study demonstrated that JNK inhibitors significantly reduced the intracellular lipid accumulation in mature adipocytes suggesting that the activation of JNK is linked with expressing adipocyte profile [27]. Complementary to this, however, current results exhibited that DEQA lowered the amounts of phosphorylated p38, ERK, and JNK (Figure 4). On the other hand, a clear dose-dependency was not observed for the lower dose treatments. Overall, ability of DEQA to downregulate the MAPK pathway suggests that it affects the differentiation of adipocytes through a MAPK-linked interaction.

To characterize the molecular interactions of DEQA that underlie its mechanism of action against adipogenesis in 3T3-L1 preadipocytes, the protein levels of phosphorylated AMPK and its direct substrate, ACC, were analyzed. It has been proposed that the activation of AMPK signaling pathway is imperative for the suppression of differentiation in 3T3-L1 cells by phytochemical compounds. Reports indicate that AMPK pathway takes part in stimulation of energy expenditure through lipolysis in adipocytes [13]. AMPK pathways are known to react to changes in balance of intracellular levels of AMP or the AMP to ATP ratio. Thus, preadipocytes that undergo adipogenesis demonstrate reduced levels of phosphorylated AMPK and its substrate ACC. DEQA was shown to increase the levels of activated AMPK and the elevated activation of this pathway resulted in the increased phosphorylation of its substrate, ACC (Figure 5). In addition, the increase of inactive AMPK and ACC along their activated counterparts indicated a direct involvement of the DEQA treatment in AMPK signaling of differentiating adipocytes. The current data evidently suggested that DEQA effectively downregulated adipogenesis in 3T3-L1 preadipocytes through the upregulation of AMPK and downregulation of MAPK pathways.

## 5. Conclusions

In current study, the promising effect of DEQA on inhibiting the adipogenic differentiation of 3T3-L1 preadipocytes was observed. It has been demonstrated that DEQA attenuated stimulated adipogenesis without showing any cytotoxic outcome on differentiating preadipocytes indicated by lowering the amount of accumulated lipid droplets. This effect suggestively targeted the proliferation and lipogenesis phases of the differentiating adipocytes, with DEQA which hindered the adipogenesis progression and reduced the expression of adipogenesis-specific transcription factors. The DEQA-induced deterioration of the adipogenesis is characterized with inhibited phosphorylation of the p38, ERK, and JNK as well as elevated phosphorylation of AMPK and ACC. It was suggested that DEQA interacts with the AMPK/MAPK pathway which in turn suppresses the expression of adipogenic transcription factors during adipogenesis of 3T3-L1 cells. Collectively, current study suggested that DEQA is a natural product with antiadipogenic properties.

## Data Availability

The data used to support the findings of this study are available from the corresponding author upon request.
